# Induction of Thoracic Aortic Remodeling by Endothelial-Specific Deletion of MicroRNA-21 in Mice

**DOI:** 10.1371/journal.pone.0059002

**Published:** 2013-03-18

**Authors:** Xing-Yi Zhang, Bao-Rong Shen, Yu-Cheng Zhang, Xue-Jiao Wan, Qing-Ping Yao, Guang-Liang Wu, Ji-Yao Wang, Si-Guo Chen, Zhi-Qiang Yan, Zong-Lai Jiang

**Affiliations:** Institute of Mechanobiology & Medical Engineering, School of Life Sciences & Biotechnology, Shanghai Jiao Tong University, Shanghai, China; The University of Tennessee Health Science Center, United States of America

## Abstract

MicroRNAs (miRs) are known to have an important role in modulating vascular biology. MiR21 was found to be involved in the pathogenesis of proliferative vascular disease. The role of miR21 in endothelial cells (ECs) has well studied in vitro, but the study in vivo remains to be elucidated. In this study, miR21 endothelial-specific knockout mice were generated by Cre/LoxP system. Compared with wild-type mice, the miR21 deletion in ECs resulted in structural and functional remodeling of aorta significantly, such as diastolic pressure dropping, maximal tension depression, endothelium-dependent relaxation impairment, an increase of opening angles and wall-thickness/inner diameter ratio, and compliance decrease, in the miR21 endothelial-specific knockout mice. Furthermore, the miR21 deletion in ECs induced down-regulation of collagen I, collagen III and elastin mRNA and proteins, as well as up-regulation of Smad7 and down-regulation of Smad2/5 in the aorta of miR21 endothelial-specific knockout mice. CTGF and downstream MMP/TIMP changes were also identified to mediate vascular remodeling. The results showed that miR21 is identified as a critical molecule to modulate vascular remodeling, which will help to understand the role of miR21 in vascular biology and the pathogenesis of vascular diseases.

## Introduction

MircoRNAs (miRs) are a class of ∼22 nucleotide non-coding RNAs that regulate gene expression of various kinds of cellular proteins by targeting their mRNA expression levels [1], [2]. MiRs are being recognized to be expressed in the cardiovascular system, and evidence supports a role of miRs in vascular biology and the pathogenesis of artery disease [3]. Among these miRs, MiR21 is found to participate in vascular remodeling by regulating proliferation, apoptosis and phenotype transformation of vascular smooth muscle cells (VSMCs) [4]–[8]. MiR21 is also found to modulate function of endothelial cells (ECs). Overexpressing miR21 decreased apoptosis and increased eNOS phosphorylation and nitric oxide production in ECs [9]. MiR21 expression was reduced in late-passage senescent human aorta ECs featuring reduced cell proliferation, enhanced apoptosis and inflammation and reduced eNOS [10]. These findings strongly suggest that miR21 have a crucial role in modulating EC biology.

ECs, which form the inner lining of blood vessel wall, serve important homeostatic functions in response to various chemical and mechanical stimuli [11]. Besides providing a selective barrier for macromolecular permeability, ECs can influence vascular remodeling via the production of growth promoting and inhibiting substances; modulate hemostasis/thrombosis; mediate inflammatory responses and regulate VSMC functions [11], [12]. Endothelial dysfunction may lead to pathophysiological states that contribute to the development of vascular disorders such as atherosclerosis, hypertension and thrombosis [11], [13].

Although the role of miR21 in vascular biology was well studied in vitro, little is known how miR21 modulates vascular remodeling in vivo. We hypothesize that change of miR21 expression in ECs may influence the vascular remodeling significantly. In present study, miR21 endothelial-specific knockout (KO) mice were established by using site-specific recombination Cre/LoxP systems. The thoracic aorta remodeling in vivo was evaluated and elucidated in the miR21 endothelial-specific KO mice. Understanding of the effect of miR21 on modulating vascular remodeling will help to define the molecular mechanisms underlying vascular homeostasis and the vascular pathology, as well as general cell biological processes.

## Materials and Methods

### Construction of miR21 flox/flox, Tek-Cre/Flp knockout mice and PCR identification

To generate miR21 endothelial-specific KO mice, targeting vector for generating null alleles of miR21 deletion mutation was constructed using the pBR322 vector, which contains a neomycin resistance gene driven by the pGK promoter, flanked by FRT sites. MiR21 targeting strategies were designed to replace the pre-miR sequences (391bp) flanked by loxP sites with the neomycin resistance cassette. For the targeting vector of miR21, a 3.2 kb fragment upstream of pre-miR21 and a 5 kb fragment downstream were generated as the 5′ and 3′ arms, respectively. The targeting vectors were then linearized and electroporated into 129SvEv-derived ES cells. 167 ES cell clones for targeting vector were isolated and analyzed for homologous recombination by PCR.

Two clones with a properly targeted miR21 allele were injected into 3.5-d C57BL/6 blastocysts, and high-percentage chimeric male mice were crossed to C57BL/6 females to achieve germline transmission of the targeted alleles. Heterozygous miR21 neo/+ mice were intercrossed with Flp-transgenic mice (J003800, Jackson laboratories) to remove the neo cassette, and Tek-Cre transgenic mice (J004128, Jackson laboratories) to remove miR21 in ECs. All the miR21 endothelial-specific KO mice in the study were products of intercrossing miR21 flox/flox (neo) mice.

For screening of ES cell clones positive for targeted miR21 flox/flox allele, electroporated G418-resistant ES cells were digested and analyzed by PCR. PCR primers consisted of P1–P2, P3–P4. PCR was performed with 35 cycles of 95°C, 45 seconds; 58°C, 3.5 minutes; and 72°C, 3.5 minutes, and 35 cycles of 95°C, 45 seconds; 66°C, 5.5 minutes; and 72°C, 5.5 minutes, respectively. PCR product for 5′ arm was about 3.7 kb; product for 3′ arm was about 5.8 kb.

Mouse tail tips were digested with 0.2 mg/ml proteinase K at 55°C overnight, and analyzed by PCR. That was performed with 30 cycles of 95°C, 45 seconds; 52°C, 1 minutes; and 72°C, 45 seconds for cF1-cR1, and 94°C, 30 seconds; 57°C, 1 minutes; 72°C, 1 minutes for FLP, Tek-Cre, respectively. The primers for genotyping of miR21 knockout are in Table 1.

**Table 1:** List of primers used for genotyping and real-time PCR.

Primer	Sequence
P1	5′-TCGTTCACTGGTGTCAGTACG-3′
P2	5′-GGCCTACCCGCTTCCATTGCTC -3′
P3	5′-CCGTGCCTTCCTTGACCCTGG -3′
P4	5′-CATCGTTGTTACTCACAAGAGC-3′
cF1	5′-AAATCCATGAGGCAAGGTGA-3′
cR1	5′-TTATGTATTGCCTGAGAGAGCTACC-3′
FLP-A	5′-CACTGATATTGTAAGTAGTTTGC-3′
FLP-B	5′-CTAGTGCGAAGTAGTGATCAGG-3′
Cre-A	5′-GCGGTCTGGCAGTAAAAACTATC-3′
Cre-B	5′-GTGAAACAGCATTGCTGTCACTT-3′
Cre-Internal positive control A	5′-CTAGGCCACAGAATTGAAAGATCT-3′
Cre-Internal positive control B	5′-GTAGGTGGAAATTCTAGCATCATCC-3′

This study was carried out in strict accordance with the recommendations in the Guide for the Care and Use of Laboratory Animals of the National Institutes of Health (publication no. 85–23, revised 1996). The protocol was approved by the Committee on the Ethics of Animal Experiments of Shanghai Jiao Tong University, School of Life Sciences & Biotechnology (Permit Number: 2011–015). All surgery was performed under sodium pentobarbital anesthesia, and all efforts were made to minimize suffering.

### Cell culture

ECs were isolated from mice aorta as described [14] with some modifications. The thoracic aorta from the miR21 endothelial-specific KO mice was treated by 2 mg/ml collagenase I (sigma) for 45 minutes at 37°C. ECs were cultured in DMEM supplemented with 20% FBS and antibiotics. All experiments were performed with ECs up to passage three and cultured to confluence before treatment.

### In situ hybridization

Locked nucleic acid (LNA)-modified oligonucleotide probes labeled with FITC at their 5′-ends were obtained from Exiqon. The sequences of miR21 probe and the scramble control probe for a negative control were 5′-/56-FAM/TCAACATCAGTCTGATAAGCTA-3′ and 5′-/56-FAM/GTGTAACACGTCTATACGCCCA-3′, respectively.

ECs were seeded on coverglass slides, reaching 50–70% confluence. The procedure was conducted with modification as described [15]. The cells were fixed with 4% formaldehyde for 30 minutes at room temperature, washed three times with PBS. Hybridization with the LNA probe (30 nM) was carried out at 20–22°C below the melting temperature of the probe overnight in hybridization buffer (50% deionised formamide, 0.3 M NaCl, 20 mM Tris HCL, pH 8.0, 5 mM EDTA, 10 mM NaPO4, pH 8.0, 10% Dextran Sulfate, 1X Denhardt’s solution, 0.5 mg/mL yeast RNA). After washing slides 2 times for 30 minutes each in washing buffer (50% formamide, 0.1% Tween-20, 1X SSC) at a temperature same as above, the slides were sealed using Gel Mount (Sigma).

### Immunofluorescence

The cultured ECs were fixed in 4% paraformaldehyde for 20 minutes, permeabilized with 0.4% Triton X-100 for 5 minutes, then blocked with PBS containing 1% BSA for 1 hour. Cre was stained with an anti-Cre-antibody (Novagen) and a FITC-conjugated secondary antibody (Molecular Probes). The vWF was stained with an anti-von willebrand factor-antibody (DakoCytomation) and a Rhodamine-conjugated secondary antibody (Molecular Probes). Aortas were fixed by 4% paraformaldehyde. Cre expression in the aorta was detected by en face staining. The cell nucleus was counter-stained with DAPI (Sigma).

### RNA isolation, RT- real time PCR and semi-quantitative RT-PCR

Total RNA of VSMCs from aorta was prepared by lysing the cells using Trizol reagent (Invitrogen, Carlsbad, CA). Different amount of RNA were reverse transcribed with M-MuLV reverse transcriptase (Fermentas) according to qPCR Quantitation Kit instruction (GenePharma) for miR21, U6, elastin, collagen I, collagen III and GAPDH (Table 2). Then, quantitative PCR was performed using primers and materials from GenePharma. The Ct values were used to calculate the relative fold difference in miR levels. The data were normalized to U6 or GAPDH expression levels.

**Table 2.Genes:** Genes , primer sequences for quantitative RT-PCR analysis.

Genes	Primer sequences
Elastin	5′-TGGTGCTACATGTTGGTGCT-3′ (forward)
(645bp)	5′-CAGTGTGAGGAGCCATCTGA -3′ (reverse)
COL1A1	5′-TGGAGAGAGAGGTGAACAAGG -3′ (forward)
(251bp)	5′-CATCACCCTTAGCACCATCG -3′ (reverse)
COL3A1	5′-TGGTTCTAATGGCATCAAAGG -3′ (forward)
(228bp)	5′-CCCTCAGATCCTCTTTCACC -3′ (reverse)
GAPDH	5′-ACCACAGTCCATGCCATCAC -3′ (forward)
(113bp)	5′-TCCACCACCCTGTTGCTGTA -3′ (reverse)

The semi-quantitative PCR use the same quantitation kit as above, but PCR was performed without SYBR Green as normal PCR. MiR21 primer set and U6 primer set were added at the same time to determine the relative amount of miR21 and U6. After denaturing at 95°C for 3 minutes, PCR was performed at 95°C for 30 seconds, 60°C for 30 seconds for 40 cycles.

### Measurement of blood pressure

MiR21 flox/flox (miR21 positive, as a control group) and miR21 flox/flox, Tek-Cre (miR21 endothelial-specific KO, as a knockout group) mice at 8 weeks were used for blood pressure measurement, as well as the subsequent experiments. The systolic, mean, and diastolic blood pressure were measured by a programmable sphygmomanometer (BP-98A; Softron) using the tail-cuff method as described previously [16].

### Arterial opening angle measurement

In this study we have modified the previous method to measure the arterial opening angle [17]. Briefly, the thoracic aorta was carefully dissected and placed into a Ca^2+^-free Krebs solution (composition in mM: 117.9 NaCl, 4.7 KCl, 1.2 KH_2_PO_4_, 25 NaHCO_3_, 1.2 MgSO_4_, 2.5 CaCl_2_, and 11 glucose, pH = 7.4). The length of the aorta above diaphragm was 10 mm, which was then cut into 4 equal length segments. Each segment was cut as a ring with length of 0.3–0.5 mm. Each ring was transferred to a Ca^2+^-free Krebs solution, aerated with 95% O_2_-5% CO_2_, and photographed by microscope (Olympus SZX16) in the no-load state. The cross section of each sector was photographed 30 minutes after the radial cut. Image Pro Plus 6.0 was used for opening angle analysis.

### Measurement of vascular reactivity of the isolated aortic strips

The method to determine endothelium-dependent vascular relaxation in thoracic aortic strips from mice was described previously with modification [18]. Briefly, aortas were opened longitudinally and specimens of 2 to 3 mm long aortic strips were cut and tested in transverse directions of aortas (the same direction as aortic rings). A silk thread (USP 9/0) was attached to both ends of the aortic strips, strips were mounted along the circular axis in 10 ml organ baths containing warmed (37°C) and oxygenated (95% O_2_∶5% CO_2_) Krebs solution (in mM, NaCl 137.4, KCl 5.9, CaCl2 2.5, MgCl_2_ 1.2, glucose 11.5, NaHCO_3_ 15.5, NaH_2_PO_4_ 1.2, and CaCl_2_ 2.5). Isometric tension was recorded by an isometric force transducer (RM6240C, Chengdu Instrument Factory, China) that was connected to an amplifier. A tension of 0.3 g was applied and the strips were equilibrated for 60 minutes, followed by precontraction with norepinephrine (10^−6^M) and endothelium-dependent relaxations triggered by increasing acetylcholine (ACh) concentrations were recorded (10^−8^M to 10^−5^M). In a separate set of experiments aortic strips were incubated with NG-Nitro-L-arginine Methyl Ester (L-NAME, 10^−6^M) for 10 minutes, followed by precontraction and determination of endothelium-dependent vascular function. Then non-endothelium-dependent relaxations to sodium nitroprusside (SNP) were recorded (10^−8^M to 10^−5^M).

### Aortic perfusion-fixation, morphometry and micro-structure analysis

Aortas were fixed in situ by perfusing with 0.9% sterile saline, followed by 4% paraformaldehyde at a pressure of 100 mmHg as described previously [19]. The descending 0.5 mm thoracic aorta above diaphragm was then carefully excised and bisected to two equal segments, 8 µm frozen cross sections were cut and mounted on glass slides. Sections from each group were stained for quantitative analysis with either Van Gieson or Weigert stains [20] and Haematoxylin-Eosin (H-E) stains. Microscopic images of aortic tissue sections were visualized on a microscope (Olympus BX51). All subsequent image analysis, including morphometry, was performed using IPP 6.0. Morphometry measurements were made in four areas, located on the clock at 0, 3, 6, and 9 hours, from equal bisected 6 vessel sections, which were selected from the continuous slices of each group at the same segment, and the average value of five vessels was used to determine the group average. The perimeters of the media and its thickness were measured by analyzing the H-E staining section. The contents of collagen and elastin in aorta were determined by imaging stained aortic sections at high magnification under the same condition with bright field microscopy. The percent area was calculated by the area occupied by the elastic fibres (black) or collagen fibres (Red) divided by the total area of selected media with a length of 200 µm [21]–[23].

### Pressure (P)-volume (V) relationship of thoracic aorta determination

In the center of the thoracic aorta, two markers were made on both ends of the vessel segment length of about 6 mm, and measure the distance between two points, i.e., the in vivo length of blood vessel. Bipolar coagulator was used to occlude small arteries. Then, cut the thoracic aorta out, floating wash blood in saline, and then measuring the distance between two marked points, that is the in vitro length of blood vessel. The marked blood vessel is prepared for P-V relationship determination.

A vascular mechanical property detector (VM-03, MEDENG Electronic Equipment Co., Ltd., China) was used to make physiological saline infusion to load/unload test of thoracic aorta. Firstly, vascular segments had three times load/unload for precondition. Then, three consecutive repeated load/unload and P-V relationship data were obtained.

### Determination of collagen and elastin contents in aorta

Total soluble collagens were extracted overnight by using 5 mg/ml pepsin in 0.5 M acetic acid followed the instruction from Biocolor, UK. The soluble collagens included both denatured and undenatured ones were measured by the Sircol collagen assay kit (Biocolor, UK).

The aortas were dissected and added by 750 µl of 0.25 M oxalic acid. The samples were placed into a metal heating block with the thermostat set at 100°C for 60 minutes. Then the aortic elastin content was quantified by using the Fastin elastin assay kit (Biocolor, UK) according to the manufacturer's instructions.

### Western blot analysis

Aortic lysates were prepared and analyzed by Western blot as previously described [24]–[27]. The mixture was boiled for 5 min before electrophoresis. For collagen and elastin, vascular tissues were lysed in hot RIPA Lysis Buffer (strong, Beyotime), which has strong denaturing capabilities. Then the samples were thawed and triturated in sterile TenBroeck glass tissue grinders. The proteins were denatured and broken after boiling for 10 minutes. Proteins (30 µg/lane) were fractionated by SDS-PAGE on a 12% acrylamide gel under reducing conditions and blotted onto nitrocellulose membrane (Amersham). And the membrane was incubated with goat polyclonal antibody against COL1A2 (1∶500, Santa Cruz Biotechnology), mouse monoclonal antibody against COL3A1 (1∶500, Santa Cruz Biotechnology), rabbit polyclonal antibody against elastin (1∶500, Abcam) and p-PTEN(1∶500, Cell Signaling), goat polyclonal antibody against CTGF (1∶1000, Santa Cruz Biotechnology) and GAPDH (1∶500, Santa Cruz Biotechnology), rabbit polyclonal antibody against TGF-β1, MMP-2, MMP-10 and TIMP-4 (1∶500, Bioworld technology), rabbit monoclonal antibody against PTEN(1∶500, Cell Signaling), Smad 7 (1∶500, Epitomics), Smad 2, Smad 5 (1∶500, Proteintech group), p-Smad 2 (1∶500, Signalway Antibody) and p-Smad 5 (1∶500, Epitomics), respectively.

### Statistical Analysis

Each experiment was performed at least in triplicates, and all values were expressed as mean±*SD*. The student's *t*-test was used to compare two groups. Values of *p*<0.05 were accepted as statistically significant.

## Results

### Generation of the miR21 endothelial-specific knockout mice

**Figure 1:**
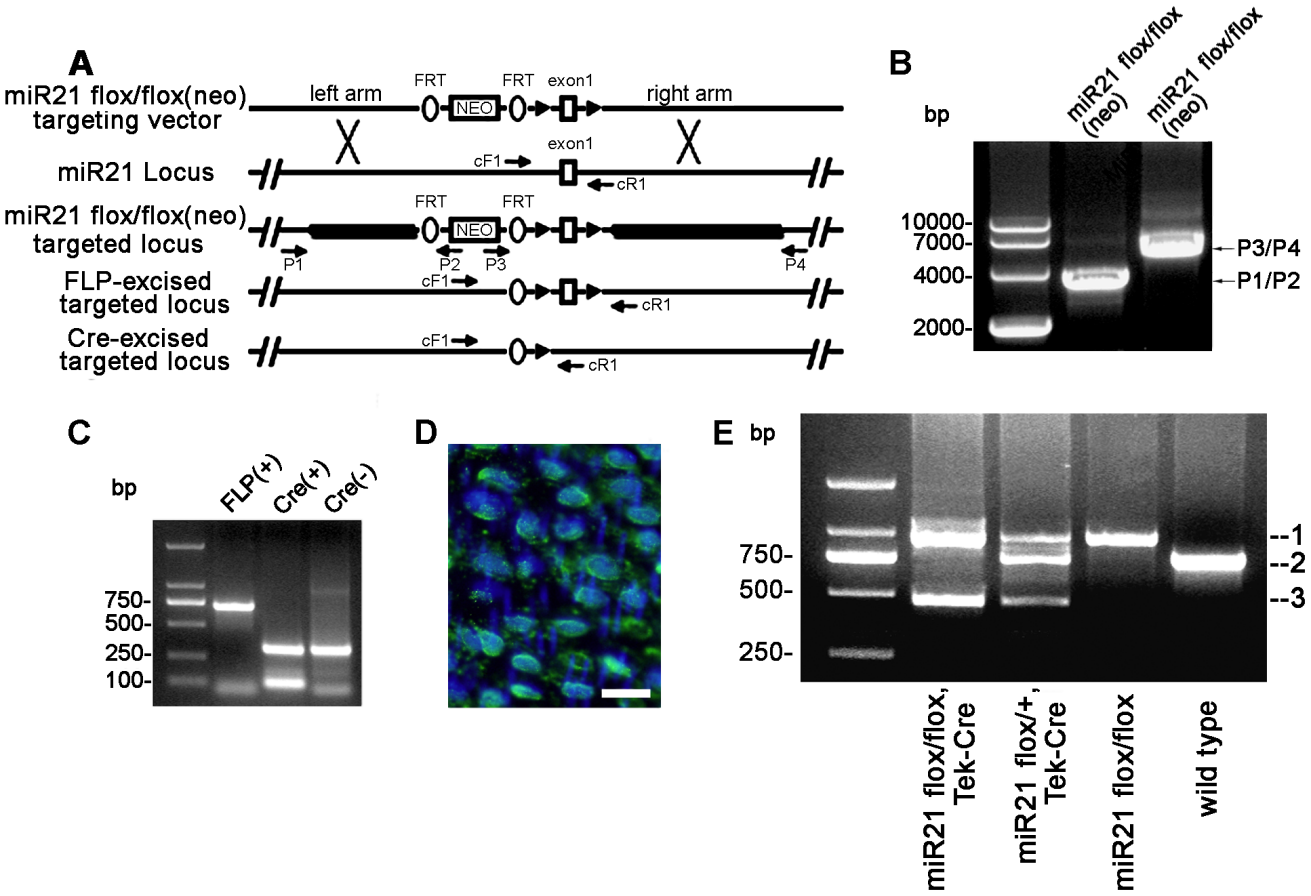
Generation of the miR21 endothelial-specific knockout mice. (**A**) Diagram for miR21 exon1-floxed targeting construct and location of primers used for genotyping of targeted locus and FLP, Cre-mediated excised targeted locus. The exon1 of miR21 and 3.2 kb, 5 kb genomic fragments flanking exon1 (left and right arm) were PCR-amplified and subcloned into a targeting vector such that exon1 of miR21 was followed by a neo cassette, and exon1 was flanked by two loxP sites, neo was flanked by two FRT sites. This targeting construct was used to generate miR21 flox/flox mice, which were crossed with FLP mice to excised neo mice, crossed with Tek-Cre mice to produce an endothelial-specific miR21 knockout. Sequences of primers are in Table 1. (**B**) Analysis of the targeting construction for the miR21 locus. Tail DNA was genotyped by PCR using P1/P2, P3/P4. The 3.2 kb and 5 kb band amplified by P1/P2, P3/P4 primer pairs indicated presence of the unexcised targeted alleles. (**C**) Analysis of the expression of FLP and Cre recombinases. PCR conditions were described in the Jackson Laboratories genotyping protocol. PCR primers are also showed in Table 1. (**D**) Merging of the DAPI and Tek-Cre immunoreactivity in the thoracic aorta of miR21 flox/flox, Tek-Cre mice. ECs were clearly stained. Bar = 25 µm. (**E**) The cF1 and cR1 primer pair identified the targeted miR21 flox/flox allele with a 925 bp product; while they amplified the wild-type allele into a 713 bp band. The cF1 and cR1 primer pair amplified the Cre-excised miR21 flox/flox locus into a 427 bp product. Thus, PCR reaction using cF1 and cR1 primer pair distinguished among miR21 flox/flox, Tek-Cre, miR21 flox/+, Tek-Cre, and miR21 flox/flox, wild type, respectively. All three PCR products (1, 2, 3) were verified by sequencing (Figure S1).

MiR21 is expressed in endothelial and all vessel support cells. To address the role of miR21 in vascular biology in vivo, mice of EC specific ablation of miR21 were established. A miR21-floxed allele was produced by inserting loxP sites in the 5′ and 3′ region of exon of mouse miR21 (Figure 1A and B). Mouse bearing targeted miR21-floxed allele was crossed with a Tek-Cre transgenic mouse line that expressed Cre recombinase specifically in EC lineage (Figure 1C and D). Offspring tail tips were genotyped by PCR using specific primers (Table 1: cF1, cR1) that allow distinction between wild-type miR21, floxed miR21, and floxed miR21 allele excised by Cre recombinase (Figure 1E). The floxed miR21 was identified as a 925 bp band marked by 1, while 713 bp band marked by 2 for wild-type miR21 and 427 bp band marked by 3 for floxed miR21 excised by Cre recombinase.

**Figure 2:**
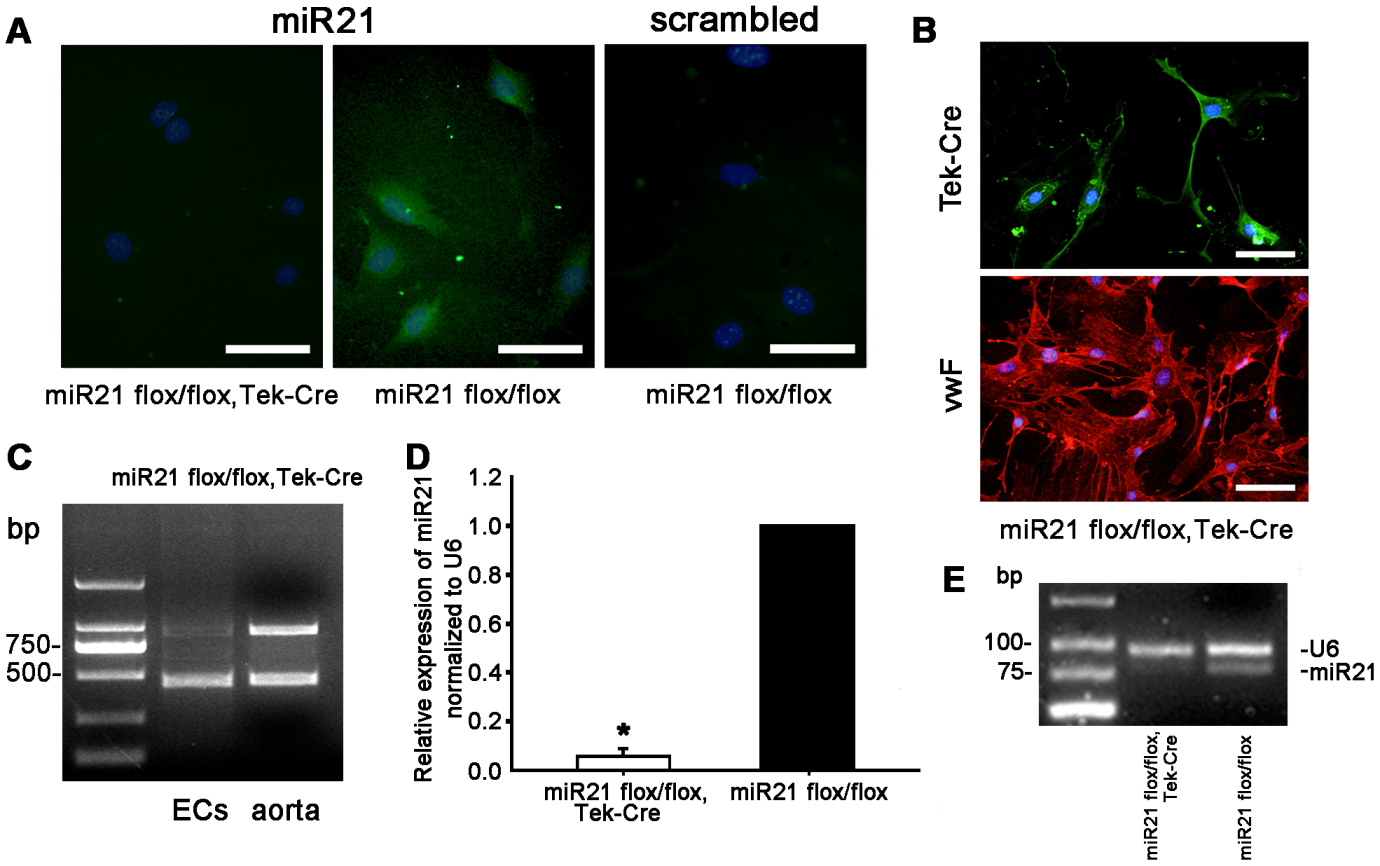
Identification of the miR21 deletion in ECs. (**A**) Aortic ECs were isolated from the miR21 endothelial-specific KO mice and cultured in growth medium. In situ hybridization(ISH) was performed to determine the expression of miR21 (Green). Results in miR21 flox/flox, Tek-Cre group (miR21 endothelial-specific KO group) showed that there was not miR21 in EC of knockout mice. The DAPI-stained nuclei are blue. Bar = 100 µm. (**B**) ECs were immunostained with Cre, vWF antibody, indicating that these ECs expressed Cre recombinase. The nuclei were stained by DAPI (Blue). Bar = 100 µm. (**C**) Analysis of excision of miR21 on DNA level in ECs and the whole aorta. It showed that miR21-floxed alleles were almost excised in ECs. (**D**) Real-time PCR was performed to determine the level of miR21 from each group described in A. The miR21 expression level of endothelial-specific KO group was significantly decreased. (**E**) Semi-quantitative RT-PCR analysis showed by mixture of miR21 and U6 primers, the expression of miR21 in the miR21 endothelial-specific KO group was significantly lower than that in the control. These two PCR products were verified by sequencing (Figure S2). Values shown are mean±*SD.*

To investigate if miR21 was deleted in ECs, in situ hybridization (ISH) was performed. Figure 2A showed that there was only minimal background staining in the miR21-knockout ECs. Immunocytochemistry demonstrated that these cells also expressed the endothelial marker vWF, and the Cre (Figure 2B). To determine if miR21 was deleted on DNA level, PCR analysis was performed and demonstrated that miR21 was almost deleted in ECs on DNA level but it existed in the aorta (Figure 2C). Real time RT-PCR results showed that expression of miR21 in ECs of the knockout group (miR21 flox/flox with Tek-Cre expression, miR21 endothelial-specific KO) was significantly lower when compared to the control group (miR21 flox/floxed without Cre expression, miR21 positive) (Figure 2D). Semi-quantitative RT-PCR analysis on the same set of samples demonstrated similar results to real time PCR results (Figure 2E). These data demonstrated that miR21 has been endothelial-specifically deleted in the miR21 mutant mice.

### Loss of miR-21 resulted in diastolic pressure dropping of the miR21 endothelial- specific KO mice

**Figure 3:**
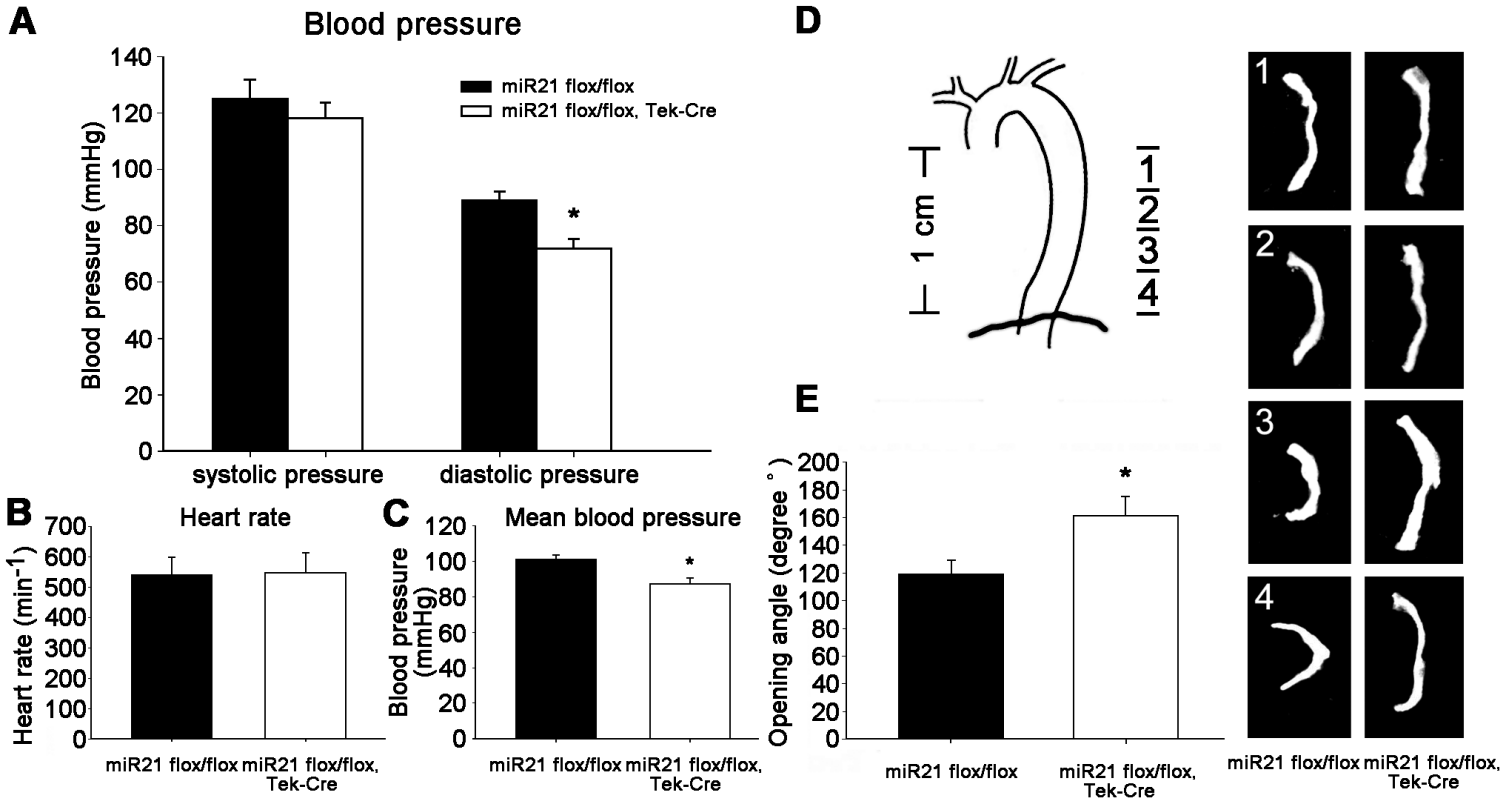
In vivo blood pressure and change of the opening angle of the arteries. (**A**) There were no difference in systolic pressure between the knockout group and the control group, but diastolic pressure was significantly reduced in the miR21 endothelial-specific KO mice. *n* = 6 for each group, *p<0.01. (**B**) No difference in heart rate was observed between the miR21 endothelial-specific KO group and control group. (**C**) Mean blood pressure was also decreased significantly in these two groups. *n* = 6 for each group, *p<0.05. (**D**) The longitudinal variation of the opening angle for four segments above diaphragm. The opening angle of aortic rings was significantly different. (**E**) The mean values of this four segments between the miR21 endothelial-specific KO group and control group were also significant different. *n* = 6 for each group, *p<0.05. Values shown are mean±*SD*.

Blood pressure was measured by a programmable sphygmomanometer using the tail-cuff method to avoid potential artifacts caused by anesthesia. As showed in Figure 3A, diastolic blood pressure was significantly reduced to 71.9±3.5 mmHg in the miR21 endothelial-specific KO mice, while the systolic blood pressure was not altered. We also detected a reduced mean blood pressure (87.4±3.1 mmHg) in miR21 endothelial-specific KO group to enhance our results about diastolic pressure reduction, but the heart rate did not change (Figure 3B and C). The present results indicated that the miR21 endothelial-specific KO results in vascular function remodeling.

### MiR21 deletion increased opening angle of thoracic aorta in the miR21 endothelial -specific KO mice

One of indexes of vascular biomechanical remodeling is zero-stress state change of a blood vessel, which can be characterized by an opening angle, defined as the angle subtended by two radii connecting the midpoint of the inner wall [28]. The longitudinal variation of the opening angle at the length of 1cm above diaphragm was shown in Figure 3D. And the mean values between the control (119.1±10.1°) and knockout group (161.3±13.9°) were found to be statistically different (Figure 3E). These data demonstrated that the miR21 endothelial-specific KO induces an increase in the opening angle of the zero-stress state, which indicates that aorta undergo vascular remodeling.

### Maximal tension and ACh-induced endothelium-dependent relaxation of aorta were impaired in the miR21 endothelial-specific deletion mice

**Figure 4:**
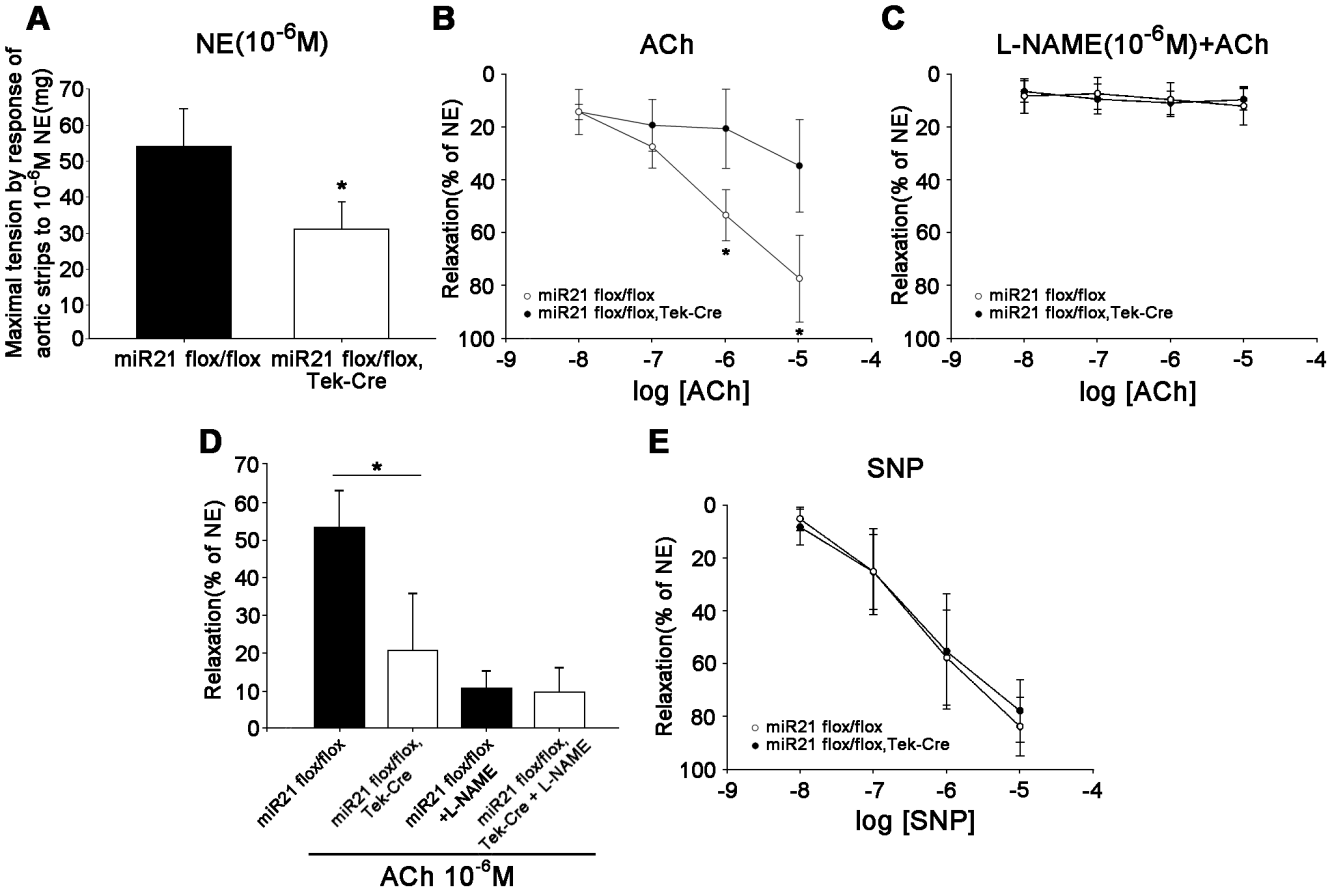
The altered aortic function of the miR21 endothelial-specific KO mice. (**A**) The response of maximal tension of aortic strips to 10^−6^M NE reduced significantly in the miR21 endothelial-specific deletion mice. *n* = 10, *p<0.05. (**B**) The amplitude of ACh-induced endothelium-dependent relaxations was significantly altered in aortic strips from the miR21 KO group compared with the control, when expressed in percent of NE-induced contraction. *n* = 6 for each group, *p<0.05. (**C**) In both the miR21 KO mice and the control, ACh-induced relaxations were abolished upon eNOS inhibition with L-NAME. *n* = 6 for each group. (**D**) The endothelium-dependent relaxations were significantly lowered by 40–50% in aortic strips from the miR21 KO mice. *n* = 6, *p<0.05. (**E**) There was no significant difference in the endothelium-independent relaxations to SNP between the miR21 KO mice and the control. *n* = 6 for each group. Values shown are mean±*SD*.

The maximal tension of aortic strips by response to norepinephrine was reduced significantly in the miR21 endothelial-specific deletion mice (Figure 4A). And severe endothelial dysfunction characterized by decreased sensitivity to ACh was observed by the endothelium dependent relaxation (Figure 4B and D). In both the miR21 KO mice and the control, ACh-induced relaxations were abolished upon eNOS inhibition with L-NAME (Figure 4C). While the endothelium independent relaxation response to SNP was not impaired in the KO group (Figure 4E). The results suggested that the contractile response to norepinephrine and the ability of the endothelium to produce nitric oxide are impaired, but there are no changes in the VSMC ability to respond to nitric oxide in the miR21 endothelial-specific knockout mice.

### Aortic morphometry

Aortic morphometry revealed an increase in thickness of thoracic aorta media. The inner diameter of thoracic aorta of miR21-KO group was significantly decreased and the wall thickness/inner diameter ratio of that was significantly increased. But the outer diameter was not significantly changed (Table 3). The morphometry results showed that wall thickness of thoracic aorta media is increased by vascular remodeling resulting from the miR21 deletion in ECs.

**Table 3:** Morphomety of the thoracic aorta of miR21 flox/flox and miR21 flox/flox, Tek-Cre mice.

	miR21 flox/flox	miR21 flox/flox, Tek-Cre
Inner semidiameter( µm)	455.12 (8.14)	438.99 (15.52)*
Outer semidiameter( µm)	485.04 (13.41)	481.55 (16.42)
Mean semidiameter( µm)	470.08 (10.60)	460.27 (15.87)
Wall thickness( µm)	27.42 (2.53)	40.45 (3.39)*
Ratio of wall thickness to inner diameter	0.030 (0.003)	0.046 (0.005)*

Values are mean *(SD)*, *n* = 5 for each group, * *p*<0.05, miR21 flox/flox vs. miR21 flox/flox, Tek-Cre by Student's unpaired *t* test.

### MiR21 deletion decreased the contents of elastin and collagen in thoracic aorta of the miR21 endothelial-specific KO mice and the stiffness

**Figure 5:**
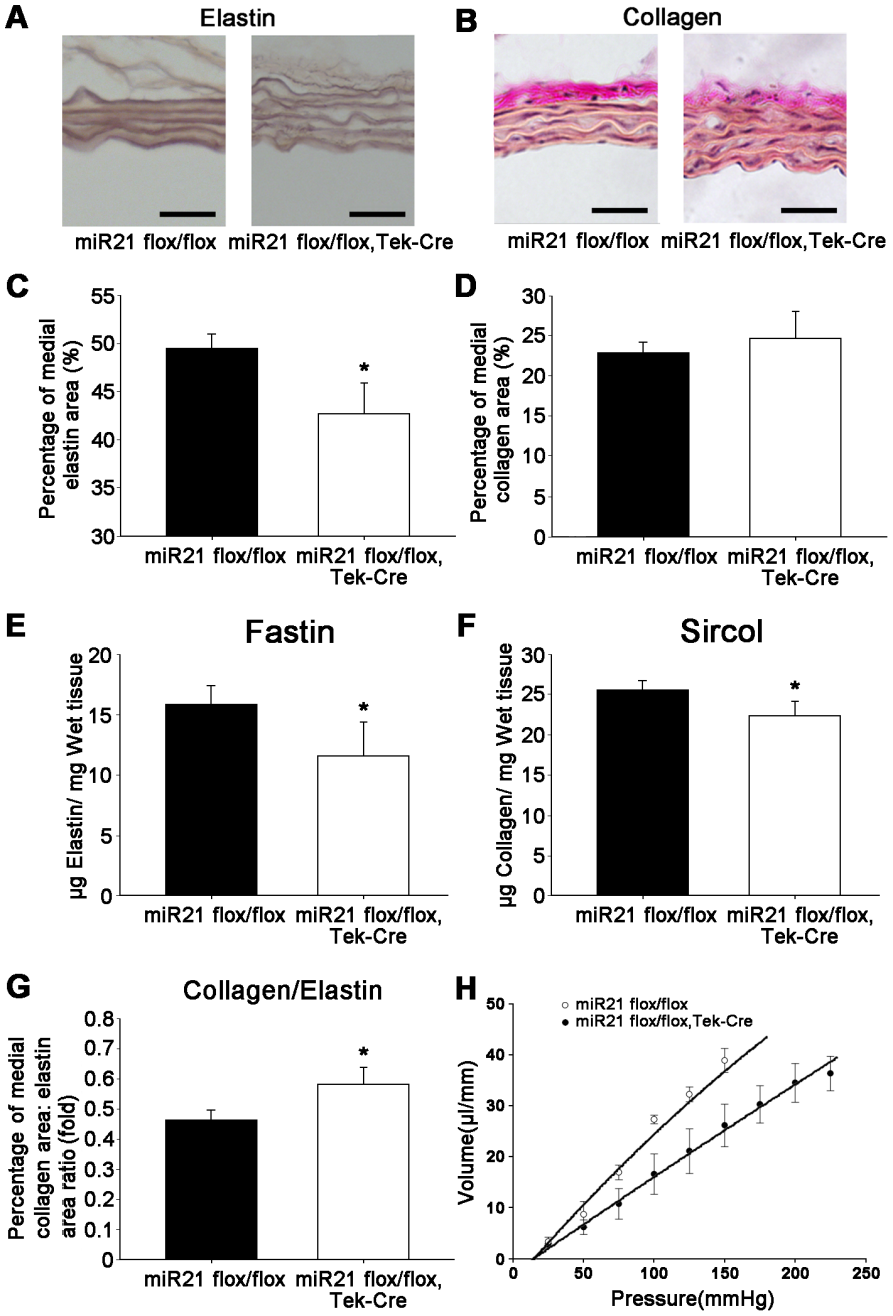
Aortic morphometry, micro-structure and contents of elastin and collagen, aortic stiffness and pressure (P)-volume (V) relationship. (A) Representative Weigert stained aortic sections showing elastin changes between the knockout group and the control group. Bar = 25 µm. (B) Representative Van Gieson stained aortic sections showing collagen changes between genotypes. Bar = 25 µm. (C) The medial area fraction of elastin content was quantitated, and significantly decreased in the miR21 endothelial-specific KO mice compared with control mice. *n* = 5 for each group, *p<0.05. (D) There were no significant changes in collagen content observed in the area percent. *n* = 5 for each group. (E) Elastin contents was evaluated by a Fastin elastin assay and found decreased in the KO group. *n* = 4 for each group, *p<0.05. (F) Collagen contents in thoracic aorta was evaluated by a Sircol collagen assay and found reduced in the KO group. *n* = 4 for each group, *p<0.05. (G) Aortic stiffness was approximated by calculating the ratio of area percentage between collagen (D) to elastin (C), which showed an elevated trend. *p<0.05. (H) P-V relation of aorta between the control and KO group. P-V relation curve of control is shifted downward to the right curve of the KO group, which showed decreased volume and increased pressure by decreasing compliance. *n* = 6 for each group. Values shown are mean±*SD*.

Aortic tissue sections from the control and knockout groups were stained with Weigert's resorcin-fuchsin for elastin. The percentage of staining area for elastin was quantitated by Image Pro Plus 6.0. The results revealed a decrease of the percentage in the miR21 endothelial-specific KO mice (Figure 5A and C). The results demonstrated that miR21 deletion decreases the content of elastin of thoracic aorta in the miR21 endothelial-specific KO mice, which implies that the flexibility of blood vessel wall is reduced after the deletion of miR21 in ECs.

Total collagen was stained with Van Gieson method and measured in the medial regions of aortic tissue sections by Image Pro Plus 6.0 to identify its percentage of staining area. No changes in collagen staining area were observed in the miR21 endothelial-specific KO group (Figure 5B and D).

The elastin content of aorta was reduced significantly in the miR21 endothelial-specific KO group (Figure 5E). The elastin content was 11.6±2.8 µg/mg of the wet weight of the vessel in the miR21 endothelial-specific KO group, while the elastin content of the control group was 15.8±1.5 µg/mg. The Sircol collagen assay results showed a similar trend as the elastin (Figure 5F). The aortic collagen content was 22.4±1.7 µg/mg of the wet weight of the vessel in the miR21 endothelial-specific KO group, while the collagen content of the control group was 25.8±0.9 µg/mg.

The ratio of collagen contents to elastin contents is calculated to estimate aortic stiffness [19]. As the ratio is higher, the stiffness is higher. The miR21 endothelial-specific KO group showed a significant increase in aortic stiffness (Figure 5G). It indicated that the mechanical properties of flexibility and stiffness are changed by the constituents alteration caused by vascular remodeling.

### The P-V relationship of thoracic aorta

The ability of a blood vessel wall to expand and contract passively with changes in pressure is an important function of large arteries and veins. This ability of a vessel to distend and increase volume with increasing transmural pressure is quantified as vessel compliance, which is the change in volume divided by the change in pressure. When miR21 was knocked out in ECs, the P-V relationship of thoracic aorta downward shifted in the compliance curve as shown in Figure 5H. The result suggested that the compliance of arota is reduced by the miR21 deletion in ECs.

### MiR21 deletion changed expressions of elastin, collagen I and collagen III in aorta of the miR21 endothelial-specific KO mice at 21 days and 8 weeks after birth

**Figure 6:**
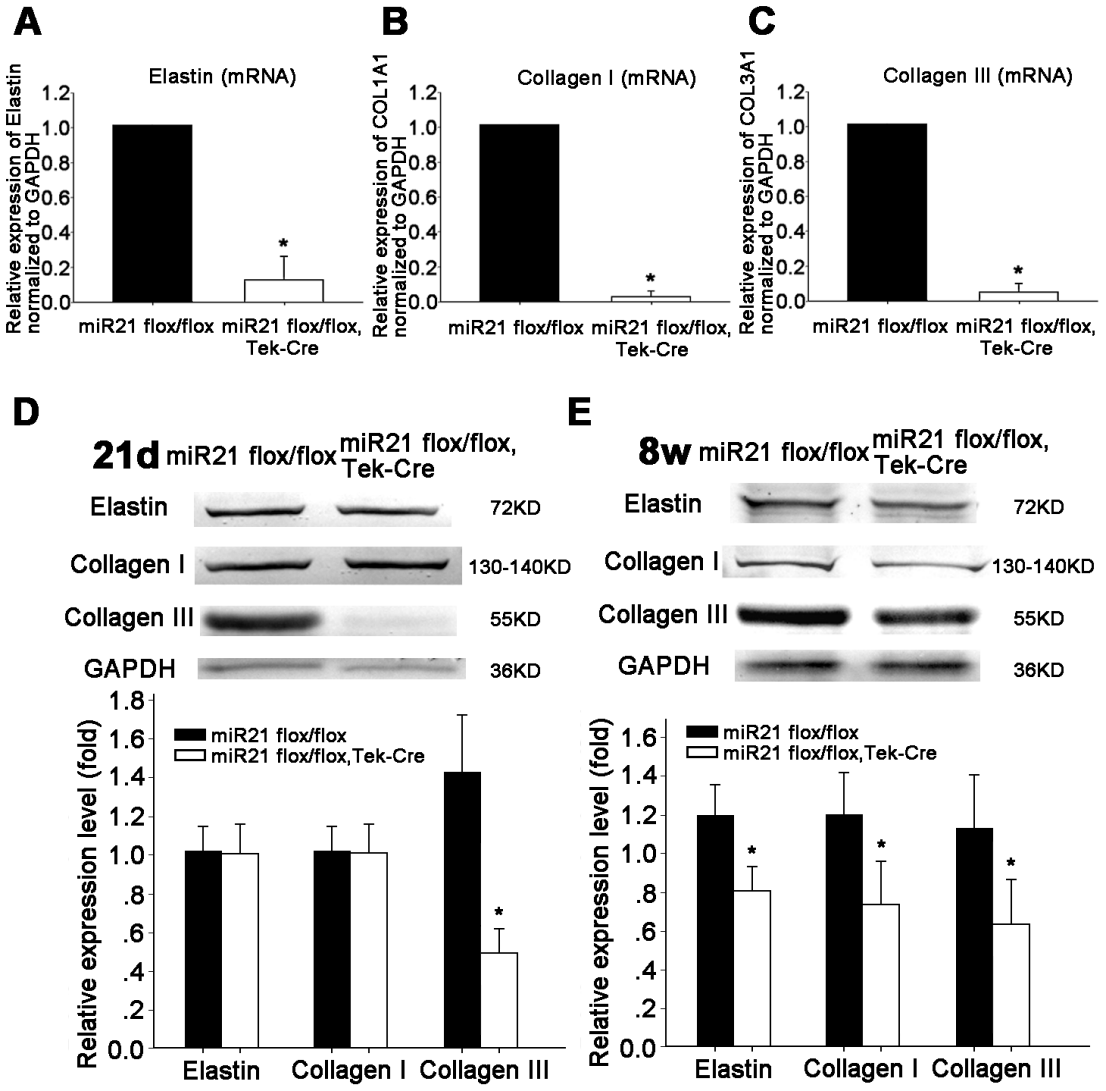
Effects of miR21 deletion on ECM expression in aorta of the miR21 endothelial-specific KO mice. (**A**) Elastin mRNA is reduced in the KO group. *n* = 5 for each group, *p<0.05. (**B**) Collagen I mRNA is significantly decreased in the KO group. *n* = 5 for each group, *p<0.05. (**C**) Collagen III mRNA is also significantly reduced in the KO group. *n* = 5 for each group, *p<0.05. (**D**) Western analysis detected the expression of elastin, collagen I, collagen III, in aorta of miR21 flox/flox, Tek-Cre (miR21 endothelial-specific KO) and miR21 flox/flox (control) mice at 21 days. Elastin, collagen I showed unchanged, but collagen III significantly reduced. *n* = 7 for each group, *p<0.05. (**E**) Western analysis detected the expression of elastin, collagen I, collagen III, in aorta of miR21 flox/flox (control) and miR21 flox/flox, Tek-Cre (miR21 endothelial-specific KO) mice at 8 weeks. Densitometry showed a decreasing trend. *n* = 7 for each group, *p<0.05. Values shown are mean±*SD*.

To confirm the histological staining results, RT real time PCR and western blot test were used. The results showed that expressions of these ECMs, elastin, collagen I and collagen III, on mRNA level or on protein level, were decreased in the miR21 endothelial-specific KO group (Figure 6A, B, C and E). The expressions of elastin and collagen were consistent with the results mentioned-above. The western results showed that the expression of collagen III was down- regulated to a low level at 21 days after birth in the miR21 endothelial-specific KO group (Figure 6D).

### MiR21 deletion affected the expression of Smad2/5/7 in aorta of the miR21 endothelial-specific KO mice

**Figure 7:**
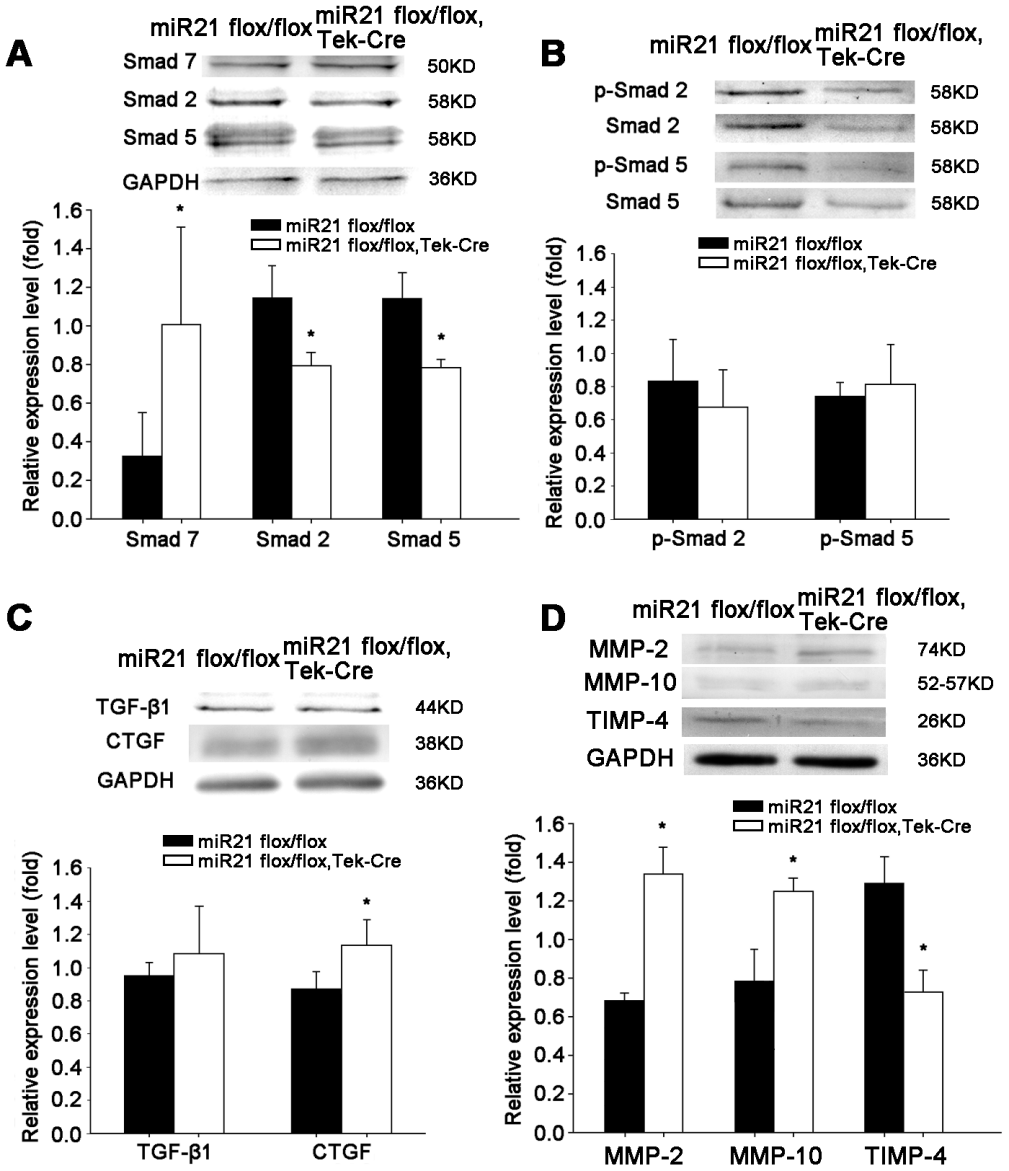
Effects of miR21 deletion on Smads, TGF-β1, CTGF, p-Smads and MMP/TIMP expressions in aorta of the miR21 endothelial-specific KO mice. (A) Western analysis detected expression of Smad2/5/7 in aorta of the miR21 endothelial-specific KO mice and control mice. Smad7 showed an increasing trend in the KO group, while Smad2 and Smad5 significantly reduced. *p<0.05, *n* = 4 for each group. (B) P-Smad2 and p-Smad5 showed no significantly difference. *n* = 5 for each group. (C)Western analysis detected expression of TGF-β1 and CTGF in the aorta of the miR21 endothelial-specific KO mice and control mice. TGF-β1 showed unchanged, but CTGF elevated significantly. *p<0.05, *n* = 5 for each group. (D) Western analysis detected expression of MMP-2, MMP-10 and TIMP-4 in aorta of the miR21 endothelial-specific KO mice and control mice. MMP-2 and MMP-10 showed an increasing trend in the KO group, while TIMP-4 significantly reduced. *p<0.05, *n* = 4 for each group. Values shown are mean±*SD*.

The expression of Smad protein family, Smad7, having been reported as a target of miR21[29], and Smad2, which reported as a negative regulating factor for miR21[30], Smad5, which mediated induction of both pre- and mature miR21[31] were all examined. As shown in Figure 7A, the expression of Smad7 was up-regulated with deletion of miR21. But the expressions of Smad2 and Smad5 were down-regulated. The results suggested that Smad proteins may mediate vascular remodeling induced by miR21 deletion. Figure 7B revealed that p-Smad2/5 levels were unchanged in blood vessels. Then the expressions of TGF-β1 and connective tissue growth factor (CTGF) were analyzed, which related to fibrosis, and regulated by smad7. Figure 7C showed that expression of TGF-β1 was unchanged but CTGF was elevated significantly in the miR21 endothelial-specific KO mice. ECM synthesis can be stimulated by pro-fibrotic cytokines such as CTGF. The ECM can be degraded by matrix metalloproteinases (MMPs), whose activity can in turn be inhibited by the endogenous tissue inhibitors of matrix metalloproteinases (TIMPs), CTGF has been shown to be a stimulator of collagen synthesis and an inducer of TIMP expression [32]. Here, we detected the expression of several MMPs/TIMPs, and found that MMP-2, MMP-10 were elevated in the miR21 endothelial-specific KO mice significantly, but TIMP-4 was reduced as the results of ECM expression (Figure 7D).

## Discussion

In the present study, we found that miR21 specific deletion in ECs modulates vascular remodeling. After establishing miR21 endothelial-specific KO mice, the blood pressure was observed and the opening angle, compliance and micro-structure of the thoracic aorta were assessed to ascertain the vascular remodeling. The data also showed that Smad7 as well as CTGF and downstream MMP/TIMP are involved in the effects of miR21 deletion in ECs. Taken together, these results suggested that miR21 in ECs plays a critical role in mediating the vascular remodeling.

We generated this knock-out mouse model and examined the expression of miR-21 at different levels. To determine whether miR-21 was deleted in ECs, PCR (Figure 2C), and ISH (Figure 2A), RT-real time PCR (Figure 2D), Semi-quantitative RT-PCR (Figure 2E) were performed to prove that miR-21 was deleted on both DNA and RNA level in ECs. These results demonstrated that the miR21 endothelial-specific KO mice are successfully established, and the mice model can be used to view the function of miR-21 in endothelial in vivo.

In this study, the blood pressure, opening angle of aortic rings, aortic histological changes, aortic compliance and expressions of ECM proteins were examined to estimate the vascular remodeling on different levels. The results showed that the diastolic pressure is significantly reduced in the miR21 endothelial-specific KO mice. Diastolic pressure is influenced by arterial or arteriolar alterations. The increase of stiffening of the large arteries can also induce a decrease in diastolic pressure [33]. In the miR21 endothelial-specific KO mice, the aortic opening angles are elevated significantly with decreasing elastin content, indicating increased residual stress in elastin-insufficient vessels [34], [35].

Structural remodeling of arteries results in altered vascular geometry. In pathological remodeling, wall thickness increases in an attempt to reduce circumferential wall stress [36], while circumferential wall stress is increased with elastin insufficiency. And the increasing of wall thickness-inner diameter ratios is a valid parameter to represent vascular abnormalities [37], [38]. Aortic morphometry also showed an increase in thickness of ascending aorta media. But the left/right common carotid artery thickness showed no significant change (Figure S3). The present study revealed that aortic elastin content in the miR21 endothelial-specific KO mice is decreased when normalized to area. In addition to the changes showed by histological staining, Sircol collagen assay, Fastin elastin assay and Western blotting were used to confirm the changes in structural protein contents. The results showed a decreasing trend in the miR21 endothelial-specific KO mice, which also matched with the Western blotting results of MMP/TIMP (Figure 7D). The mRNA expression levels of elastin, collagen I and collagen III also were decreased in the KO mice. And Western blotting results indicated that expressions of elastin, collagen I and collagen III, are all down-regulated after 8 weeks.

In the weeks after birth, arterial growth, this period involves a very rapid aortic elastin and collagen accumulation, independent of blood flow changes. Arterial pressures in near-term fetuses are about 45 mmHg, whereas pressure has risen to 65 mmHg at 3 weeks of age [39]. The rates of accumulation of elastin and collagen are greatly enhanced in the week surrounding birth in both segments of the aorta. This phase of rapid tissue accumulation preceded postnatal increases in arterial pressure [40]. Here we found that expression of collagen III was down-regulated on 21 days after birth, which implied that miR21 may relate to collagen III directly in vascular remodeling process. Collagen III in aortic wall increases the flexibility of the collagen fibrils [41].

Because aortic compliance and resiliency depend on both elastin and collagen contents, the aortic stiffness is likely affected in response to alterations in the elastic architecture [19]. We calculated the ratio of collagen content to elastin content to estimate aortic stiffness, revealing the increased stiffness (Figure 5G). Arterial compliance is a measurement of the elastic properties of a blood vessel. The stiffness index, conceptually, is the inverse of compliance [42]. Compliance here represents static compliance that is generated by expanding a vessel by a known volume and measuring the change in pressure at steady-state. Our result showed the downward shift in the compliance curve after miR21 deletion (Figure 5H), which means the compliance of aorta is reduced.

We also did the endothelial relaxation assay to interpret the relation between the low diastolic pressure and the stiffness of blood vessel. The results showed that the maximal tension of aorta was significantly reduced and the endothelium-dependent relaxation of aorta was also impaired in the miR21 endothelial-specific deletion mice. It was reported that overexpressing miR21 increased eNOS phosphorylation and nitric oxide production in ECs [9] and this result is corresponding with our endothelial relaxation result. Although the low diastolic pressure is also likely to be caused by other factors such as smooth muscle cells and/or fibroblast, rather than ECs, the maximal tension of aorta is reduced significantly in the miR21 endothelial-specific deletion mice may interpret the reason for low diastolic pressure.

When miR21 is knocked out, Smad7 is increased significantly in our results. Over expression of Smad7 inhibits TGF-β1 induced extracellular matrix expression [43]. To determine if miR21 directly regulated only Smad7 in Smads protein family, Smad 2, Smad 5 were also examined. Their expression showed a reversed trend in Smad7 (Figure 7A). Smad2 is specific mediator of TGF-β/activin pathways, whereas Smad5 is involved in bone morphogenetic protein signaling [44], which contributes to abnormal smooth muscle cell proliferation [45]. Thus, miR21 may mediate vascular remodeling mainly through Smad7.

Smad7 is an inhibitory Smad that regulates TGF-β1 signaling. CTGF is recognized as a potent downstream mediator of the fibrogenic effects of TGF-β1 [46], [47]. Our results showed TGF-β1 unchanged, while CTGF level is elevated in the miR21 endothelial-specific KO mice, which may cause the proliferation of connective tissue cells [48] and VSMCs, and influence the expression of ECM by MMP/TIMP expression [49], [50]. We detected several MMPs and TIMPs, and found that MMP-2 and MMP-10 are elevated, but TIMP-4 is reduced in the miR21 endothelial-specific KO mice, which demonstrated a decreased expression of ECM correlate with the results of elastin and collagen.

In the cultured ECs, the expression of Smad7 is up-regulated with deletion of miR21. But the expressions of Smad2 and Smad5 were down-regulated (Figure S4A). The expression of TGF-β1 was unchanged but CTGF was decreased in the miR21-deletion ECs, which showed the same trend as reported [46] (Figure S4B). Figure S4C revealed that p-Smad5 levels increased in the cultured miR21-deletion ECs. Smad5 is involved in bone morphogenetic protein signaling [44], which contributes to abnormal smooth muscle cell proliferation [45].

As a conclusion, the present study identified miR21 as a critical molecule to modulate vascular remodeling process. Its limitation is that although a knockout model is established and studied in vivo, we can not elucidate the underlying molecular mechanisms how miR21 knocked out in ECs induces vascular remodeling. These issues will be addressed in our future studies. The results will help to improve our understanding of the effects of miR21 in vascular biology and the pathogenesis of vascular diseases.

## Supporting Information

Figure S1 **Examining PCR products in** **Figure 1E** **by Sequencing.**
(TIF)

Figure S2 **Examining PCR products in** **Figure 2E** **by Sequencing.**
(TIF)

Figure S3 **Morphomety of the thoracic aorta, ascending aorta and carotid artery of miR21 flox/flox and miR21 flox/flox, Tek-Cre mice.**
(TIF)

Figure S4 **Effects of miR21 deletion on Smads, TGF-β1, CTGF, p-Smads expression in ECs from the miR21 endothelial-specific KO mice and the control.**
(TIF)
