# Supplementary figures and images for: Induction of Thoracic Aortic Remodeling by Endothelial-Specific Deletion of MicroRNA-21 in Mice
Source: PLoS One. 2013 Mar 18;8(3):e59002. doi: 10.1371/journal.pone.0059002 (PMC3601125; doi:10.1371/journal.pone.0059002)

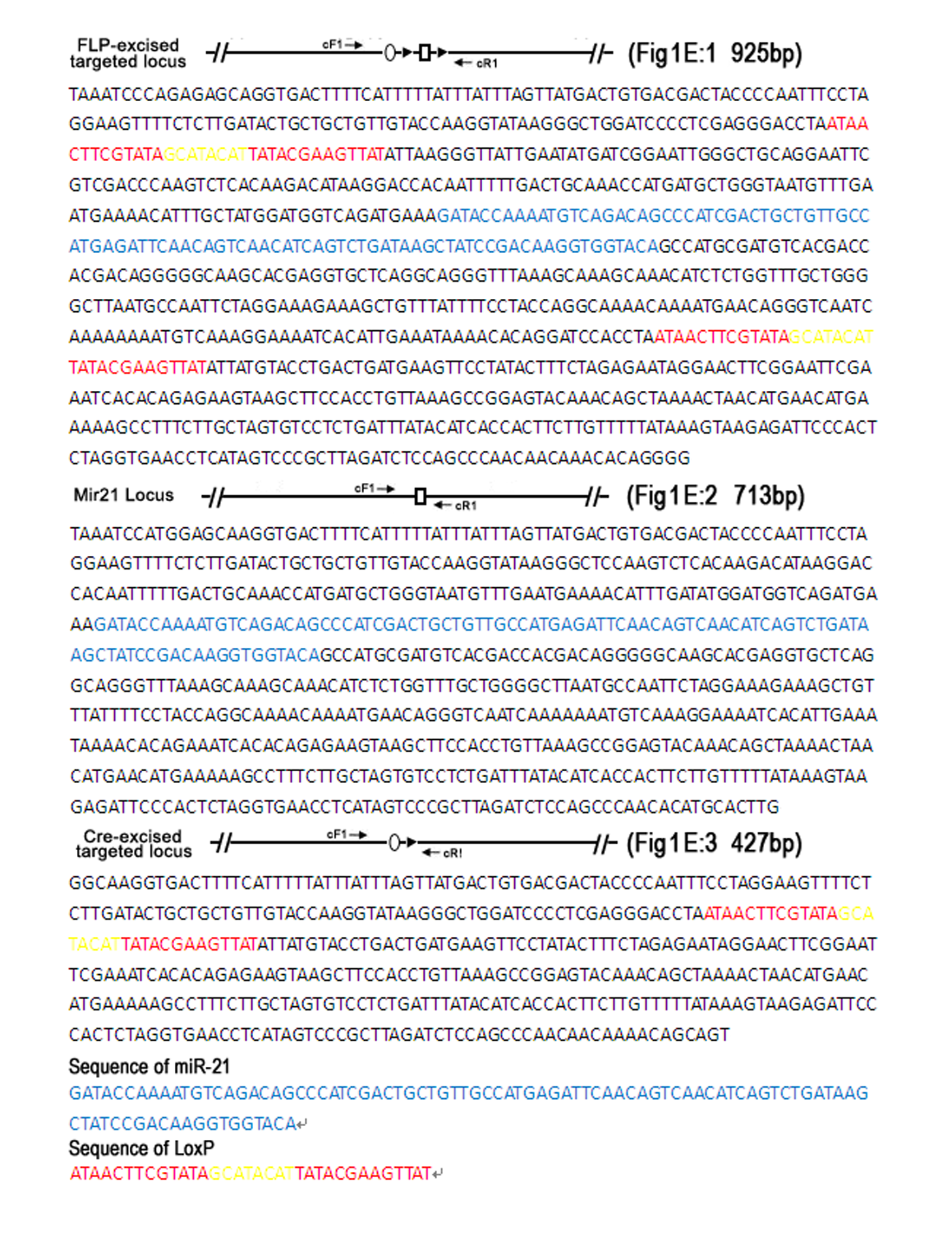

Supplement: Figure S1 — Examining PCR products in Figure 1E by Sequencing. (TIF) [file pone.0059002.s001.tif]

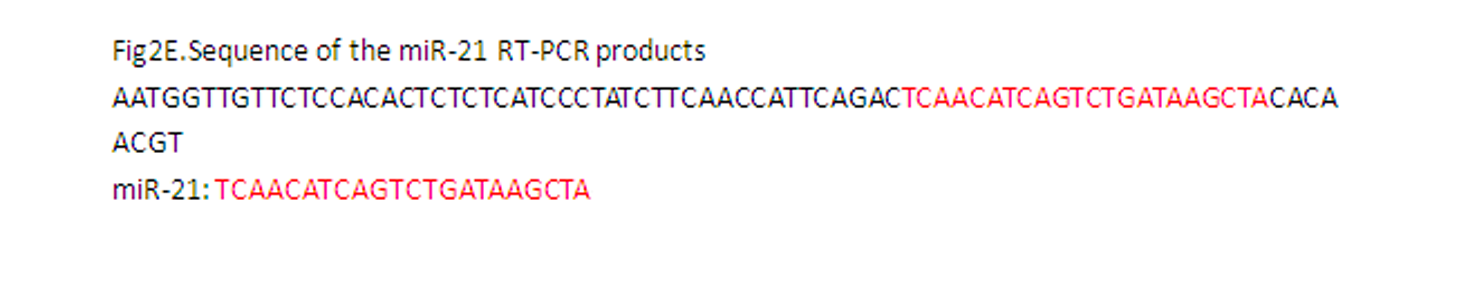

Supplement: Figure S2 — Examining PCR products in Figure 2E by Sequencing. (TIF) [file pone.0059002.s002.tif]

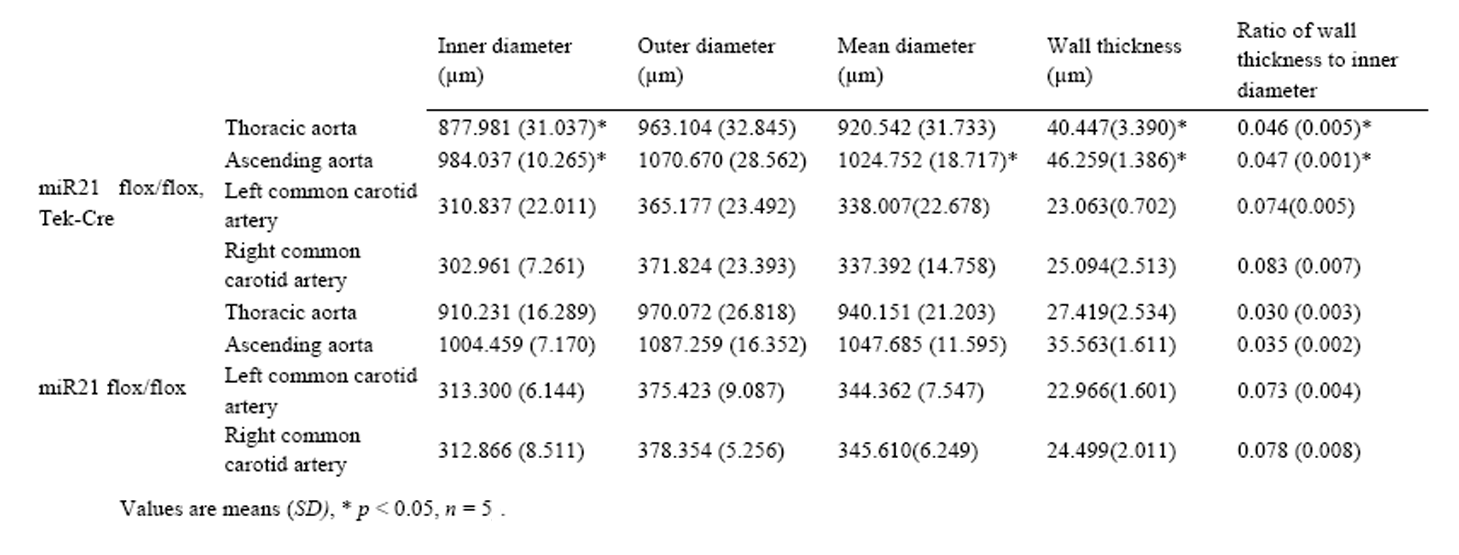

Supplement: Figure S3 — Morphomety of the thoracic aorta, ascending aorta and carotid artery of miR21 flox/flox and miR21 flox/flox, Tek-Cre mice. (TIF) [file pone.0059002.s003.tif]

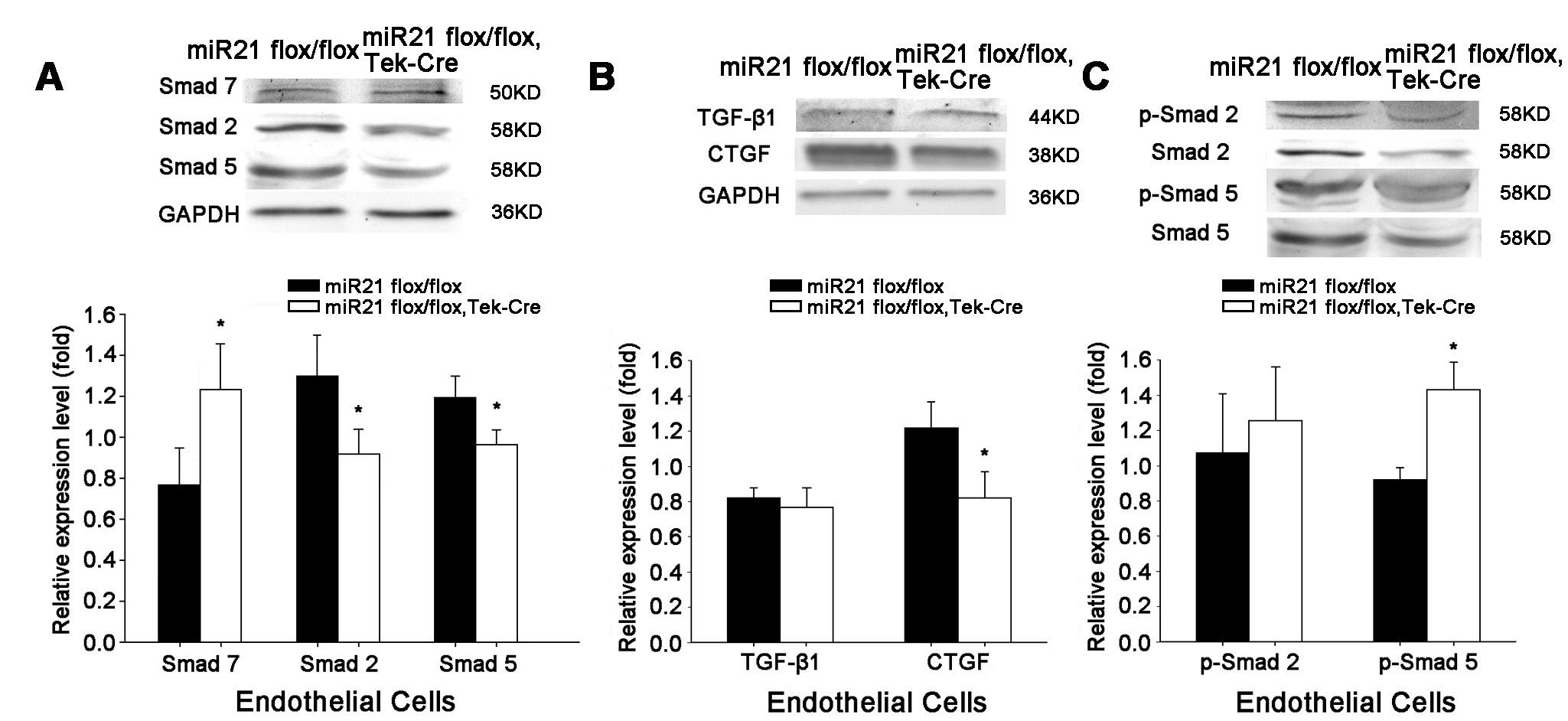

Supplement: Figure S4 — Effects of miR21 deletion on Smads, TGF-β1, CTGF, p-Smads expression in ECs from the miR21 endothelial-specific KO mice and the control. (TIF) [file pone.0059002.s004.tif]
